# Prevalence and risk factors of vitamin D deficiency among Afghan primary school children

**DOI:** 10.1038/s41598-024-77330-9

**Published:** 2024-11-08

**Authors:** Bilal Ahmad Rahimi, Aziz Ahmad Khalid, Asmatullah Usmani, Wahid Ahmad Khalid, Abdul Qadeer Baseer, Javed Ahmad Rahimi, Walter R Taylor

**Affiliations:** 1 Department of Pediatrics, Faculty of Medicine, Kandahar University, Durahi, Beside Aino Mena Town, District 10, Kandahar, Afghanistan; 2 Faculty of Medicine, Afghan International Islamic University, Kabul, Afghanistan; 3 Department of Economics, Jamia Millia Islamia, Central University, New Delhi, India; 4 Department of Biology, Faculty of Education, Kandahar University, Kandahar, Afghanistan; 5 Department of Economics, Savitribai Phule Pune University, Pune, Maharashtra India; 6 Department of Business Administration, Gujarat University, Ahmedabad, Gujarat India; 7 Mahidol Oxford Tropical Medicine Clinical Research unit (MORU), Mahidol University, Bangkok, Thailand; 8 Centre for Tropical Medicine and Global Health, Nuffield Department of Medicine, University of Oxford, Oxford, UK

**Keywords:** Vitamin D, 25(OH)D, Afghanistan, Kandahar, School children, Prevalence., Diseases, Malnutrition

## Abstract

Vitamin D deficiency is common in many societies and causes rickets and non-skeletal disorders in children. There are no published data on vitamin D deficiency in Afghanistan. We, therefore, investigated the prevalence and associated factors of vitamin D deficiency in Afghan school children in Kandahar City, Afghanistan. This cross-sectional analytical study was conducted from September 2022 to April 2023 in 510 primary school students aged 6–15 years from six randomly selected schools. Data were analyzed by using descriptive statistics, Chi-square test, and multivariate logistic regression. Of the 510 enrolled children, 54.3% were boys and 91.8% were poor. The mean serum 25(OH)D concentration was 9.3 ng/mL. Vitamin D deficiency (< 20 ng/mL) was detected in 436/510 (85.5%) children that was severe in 267/510 (52.4%). By logistic regression analysis, independent factors for vitamin D deficiency were: (i) older age group 11–15 vs. 6 − 10 years, adjusted odds ratio (AOR) 2.8 (95% confidence interval 1.2–6.2), (ii) poverty AOR 2.0 (1.0–4.3), (iii) not doing outdoor physical activity AOR 4.8 (2.8–8.1), and (iv) daily sunlight exposure < 60 min AOR 2.2 (1.3–3.7). Although Kandahar is very sunny throughout the year, vitamin D deficiency is highly prevalent among school boys and girls, placing them at great risk of vitamin D-deficient rickets. More work is needed to define the country-wide prevalence of vitamin D deficiency to inform robust strategies of vitamin D supplementation, the provision of vitamin D-fortified food to the school children in Kandahar City and health education programs that can be conducted with the help of international organizations.

## Introduction

Vitamin D is a fat-soluble vitamin, mostly responsible for the health and growth of bones by enhancing the absorption of calcium, magnesium, and phosphate^1^. The main source of vitamin D is exposure to sunlight resulting in the conversion of 7-dehydrocholesterol to vitamin D3 (cholecalciferol) in the skin^2^, which undergoes two metabolic steps: C-25 hydroxylation in the liver to 25 hydroxyD3 (25-hydroxycholecalciferol) and then to 1,25 dihydroxyD3 (1,25 dihydroxycholecalciferol in the kidneys. Vitamin D in food is in the form of vitamin D2 (ergocalciferol) and D3 and foods rich in vitamin D3 include oily fish like salmon, trout, and tuna; cod liver oil is a particularly rich source^3^. Other food sources are egg yolk, fortified cereals, and fortified milk. Vitamin D2 is found in plants and mushrooms are a rich source of vitamin D2. Vitamin D2 undergoes the same metabolism as vitamin D3 and collectively they are known as 25(OH)- and 1,25(OH)_2_D. 1,25(OH)_2_D is the active form of vitamin D that promotes the active absorption of calcium in the small intestine and this is the form that is measured in serum.

The concentration of 25(OH)D reveals the overall status of vitamin D in the body and concentrations < 50 nmol/L (20 ng/mL) are considered diagnostic of vitamin D deficiency^1^. In the absence of renal dysfunction, common causes of vitamin D deficiency include poor diet, limited sun exposure, increased skin pigmentation, use of sunblock creams, and changes in latitude^1,2^. In children, severe vitamin D deficiency might cause rickets^4^ while moderate vitamin D deficiency negatively affects bone acquisition^5,6^. the prevalence of vitamin D deficiency varies by geographical region. Based on the reports of national surveys and other studies, the prevalence of vitamin D deficiency was ~ 40% in Europe (< 50 nmol/L)^7^, 23–30% in the United States of America^8,9^, 34% in Africa (< 50 nmol/L)^10^, 30–90% in the Middle East^11^, 20% in Australia^12^, and 56% in China^13^.

A study was conducted among 107 socioeconomically deprived children under five years of age in Kabul, the capital city of Afghanistan. The median concentration of serum 25(OH)D was 5 ng/mL (range 2–24 ng/mL) with no statistically significant difference between boys and girls. The serum levels of 25(OH)D were reported to be < 8 ng/mL, 8–15 ng/mL, and > 15 ng/mL in 73.1%, 23.1%, and 3.8% of the children, respectively^14^. In another study of 308 adolescents in Kabul, 61.0% (188/308) had vitamin D deficiency; 26.3% (81/308) had mild or moderate vitamin D deficiency and 34.7% (107/308) had severe vitamin D deficiency^15^. A hospital-based study was conducted in Kabul on 4000 children, aged 1 month to 18 years, who visited the pediatric department. The prevalence of vitamin D deficiency was 41.3% (1650/4000) with 22.9% (917/4000) having severe vitamin D deficiency^16^.

Very few studies have been conducted in Kandahar involving children^17–22^ and none has investigated vitamin D deficiency. In a personal communication with pediatricians in Kandahar City, the issue of increased vitamin D deficiency among Kandahar children was raised. Although there is plenty of sunshine in Kandahar throughout the year, pediatricians still observe signs and symptoms of vitamin D deficiency among school-age children. To the best of our knowledge, there has never been a study from Afghanistan that has examined the prevalence of and associated risk factors for vitamin D deficiency in primary school children. We, therefore, set out to conduct such a study in Kandahar and report the results herein.

## Materials and methods

### Study design and study area

This was a school-based, cross-sectional study conducted during a seven-month period, from September 2022–April 2023, in Kandahar, the second largest city of Afghanistan, situated in south-west Afghanistan (coordinates: 31° 37′ 12″ N 65° 42′ 57″ E) at an altitude of 1,010 m above sea level. The population of Kandahar is a little over 614,000 people and there are 145 schools located in the city.

### Study population and sample size calculation

The study population of this study was composed of schoolchildren aged 6–15 years who were permanent residents of Kandahar. Children were excluded if they had diseases of the skin, liver, kidneys, and intestine, that might affect the absorption or metabolism of vitamin D, and those who refused (either child or guardian) to participate in the study.

The sample size was calculated using the software of Epi Info version 7.2.2.6 (CDC, Atlanta, Georgia, USA). We added a 20% non-response rate. The selection of a 20% non-response rate was based on the rule already set for this study by the quality control committee of Kandahar University Research Center. So, our sample size was 565 children.

Among the 565 children, four (0.7%) had skin diseases (all five children had psoriasis), two (0.4%) had liver diseases (both had jaundice and positive history of hepatitis B), one (0.2%) had kidney disease (this patient had nephrotic syndrome), and guardians of 48 (8.5%) children did not agree to participate in the study. So, we collected data from 510 children.

### Sample collection and laboratory procedures

Six government schools (three for boys and three for girls) were selected using a lottery method of randomization. From each school, children in grades 1–5 were also randomly selected by lottery. Socio-demographic, anthropometric, and laboratory data of the enrolled children were collected using researcher-made questionnaires in the Pashto and Dari languages. Data were collected on paper case record forms by well-trained investigators who were trained by Kandahar University Faculty of Medicine researchers for three days. One day consisted of classroom training followed by two days of practical training in the selected schools.

Anthropometric data, like height and weight, were collected from all the enrolled children. They stood barefooted against a wall and their height was measured using a stadiometer with an accuracy of 0.1 cm. All children were weighed in light clothes using a digital balance of 0.1 kg. For quality control, approximately 10% of the measurements were randomly selected and measured by an experienced researcher who was blinded by the previous measurement results; the selection of 10% was based on the rule already set by the quality control committee of Kandahar University Research Center.

A total of 5 mL of blood was taken for serum 25(OH)D levels and stored at room temperature before transfer to the laboratory within one hour. Samples were centrifuged and serum 25(OH)D was measured using a chemiluminescence immunoassay (Biomerieux, Mini VIDAS, Paris, France). All the lab examinations were performed following the guidelines and regulations of the Afghanistan Ministry of Public Health. Vitamin D status was classified based on the serum level of 25(OH) D (see definitions below). All methods and procedures were performed in accordance with the relevant guidelines and regulations.

### Ethical considerations

Ethical approval was obtained from the Ethics Committee of Kandahar University (code number KDRU-EC-2022.25) as well as permission from the Kandahar Province Education Department. Before the data collection and following an explanation of the study, written informed consent and assent were obtained freely from all the parents/guardians and children. For the data collection, only the initials of the study participants were used on the case record forms and sample labeling but all collected data were coded and de-identified for data entry.

### Data entry and analysis

All the data were double entered and cleaned in Microsoft Excel 2021 and then imported into the Statistical Package for the Social Sciences (SPSS) version 22 (Chicago, IL, USA) for statistical analysis. To summarize demographic characteristics, descriptive analyses were used, such as frequency, percentage, mean, and standard deviation (SD). The chi-square test (using crude odds ratio [COR]) was used to assess the binary association between categorical variables and students’ t-test or nonparametric equivalent were used for analyzing continuous data. All statistically significant variables in the univariate analyses were assessed for independence in a multivariate logistic regression (using adjusted odds ratio [AOR]) to determine the risk factors associated with vitamin D deficiency; factors examined included age, sex, and economic status. All analyses were two-sided and a *p*-value < 0.05 was considered statistically significant.

### Definitions

**Cut-off levels of 25(OH)D for vitamin D status** ^23^

- Vitamin D sufficiency: ≥30 ng/mL.
- Vitamin D insufficiency: 20–29.9 ng/mL.
- Vitamin D mild/moderate deficiency: 10–<20 ng/mL.
- Vitamin D severe deficiency: <10 ng/mL.

**Poverty**: Family income < 170 Afghanis (< 1.90 USD) per person per day^24^.

**Physical activity**: School children and their parents/guardians were asked about the number of days in one week they did physical activity (walking, recreational activity, or sports) and were into acceptable (physical activity ≥ 3 days per week) or unacceptable (physical activity < 3 days per week).

**Fitzpatrick classification of skin types** ^25^

- Type I: Very white or very fair skin color.
- Type II: White or fair skin color.
- Type III: Medium white to light brown skin color.
- Type IV: Olive or moderate brown skin color.
- Type V: Brown to dark brown skin color.
- Type VI: Very dark brown to black skin color.

## Results

**Fig. 1:**
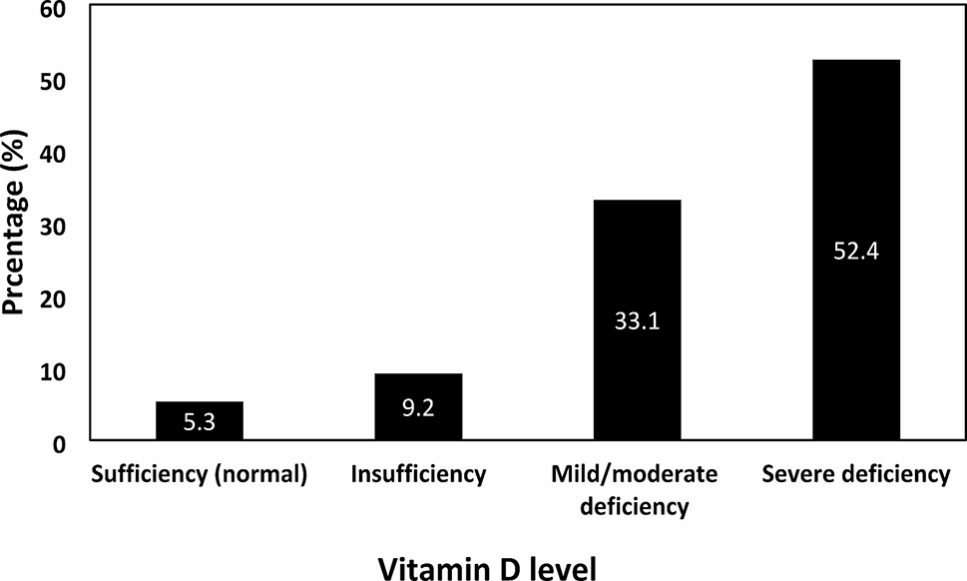
Vitamin D status (based on serum 25(OH)D) among school children in Kandahar city of Afghanistan.

Of 565 children assessed, 510 were enrolled in autumn (32.0%), winter (34.7%), and spring (33.3%); boys and girls were roughly equal. The mean (SD) age, height, and weight were 8.9 (2.3) years, 127.5 (9.4) cm, and 20.3 (3.1) kg, respectively. Overall, 401 children were aged 6–10 years and the majority ethnic group was Pashtun (Table 1). The mean (SD) level of serum 25(OH)D in all children was 9.3 (6.5) ng/mL and 436/510 (85.5%) had vitamin D deficiency [25(OH)D value < 20 ng/mL]; 267/510 (52.4%) suffered from severe (< 10 ng/mL) vitamin D deficiency (Fig. 1; Table 2).

**Table 1:** General characteristics of the enrolled boys and girls.

Variable	Total N = 510	Boys N = 277	Girls N = 233	*P*-value
Age (years)	8.9 (2.3)	9.1 (2.4)	8.4 (2.1)	0.754
Age				
6–10 years 11–15 years	401 (78.6) 109 (21.4)	204 (50.9) 73 (67.0)	197 (49.1) 36 (33.0)	0.027
Height (m)	127.5 (9.4)	128.8 (12.2)	125.1 (8.1)	0.210
Weight (kg)	20.3 (3.1)	20.9 (2.8)	19.2 (3.4)	0.841
BMI	16.4 (1.8)	17.5 (1.6)	16.1 (1.9)	0.752
Ethnicity				
Pashtun Non-Pashtun	393 (77.1) 117 (22.9)	245 (62.3) 32 (27.4)	148 (37.7) 85 (72.6)	0.018
25(OH)D (ng/mL)	9.3 (6.5)	10.5 (5.8)	8.1 (6.1)	0.091

Continuous data are mean and standard deviation (SD), categorical data are n (%).

25(OH)D, 25-Hydroxy vitamin D; BMI, Basal metabolic rate; m, meter; kg, kilogram; ng/mL, nanogram per milliliter.

The vast majority, 91.8% (468/510), of children were poor, 67.5% (344/510) did little outdoor physical activity, and only 17.6% (90/510) ate fish at least once every two weeks. Most of the study participants had either Fitzpatrick Type III (297/510 [58.2%]) or Type IV (198/510 [38.8%]) skin color while 32 (6.3%) girls only used topical sunscreen (Table 2). None of the children were taking vitamin D supplements or eating vitamin D-fortified food.

**Table 2:** Socio-demographic and other characteristics of the enrolled school boys and girls.

Variable	Total N = 510	Boys N = 277	Girls N = 233	*P*-value
Mother literacy level				
Literate Illiterate	72 (14.1) 438 (85.9)	23 (31.9) 254 (58.0)	49 (68.1) 184 (42.0)	0.015
Father literacy				
Literate Illiterate	60 (11.8) 450 (88.2)	19 (31.7) 258 (57.3)	41 (68.3) 192 (42.7)	0.013
Family economic status*				
Poor Not poor	468 (91.8) 42 (8.2)	246 (52.6) 31 (73.8)	222 (47.4) 11 (26.2)	0.050
Obesity present				
Yes No	18 (3.5) 492 (96.5)	6 (33.3) 271 (55.1)	12 (66.7) 221 (44.9)	0.095
Doing outdoor physical activity				
Yes No	166 (32.5) 344 (67.5)	145 (87.3) 132 (38.4)	21 (12.7) 212 (61.6)	0.004
Daily sunlight exposure				
< 30 min 30–59 min ≥60 min	154 (30.2) 210 (41.2) 146 (28.6)	11 (7.1) 144 (68.6) 122 (83.6)	143 (92.9) 66 (31.4) 24 (16.4)	< 0.001
Eating fish (at least once in two weeks)				
Yes No	90 (17.6) 420 (82.4)	64 (71.1) 169 (40.2)	26 (28.9) 251 (59.8)	0.013
Topical use of sunscreen				
Yes No	32 (6.3) 478 (93.7)	0 (0.0) 277 (57.9)	32 (100.0) 201 (42.1)	< 0.001
Skin color (Fitzpatrick scale)				
Type I Type II Type III Type IV Type V Type VI	0 (0.0) 12 (2.4) 297 (58.2) 198 (38.8) 3 (0.6) 0 (0.0)	0 (0.0) 4 (33.3) 110 (37.0) 160 (80.8) 3 (100.0) 0 (0.0)	0 (0.0) 8 (66.7) 187 (63.0) 38 (19.2) 0 (0.0) 0 (0.0)	0.062
Vitamin D status^†^				
Sufficient (normal) Insufficient Mild/moderate deficiency Severe deficiency	27 (5.3) 47 (9.2) 169 (33.1) 267 (52.4)	19 (70.4) 20 (42.6) 92 (54.4) 146 (54.7)	8 (29.6) 27 (57.4) 77 (45.6) 121 (45.3)	0.069
Season of data collection				
Autumn Winter Spring	163 (32.0) 177 (34.7) 170 (33.3)	87 (53.4) 90 (50.8) 88 (51.8)	76 (46.6) 87 (49.2) 82 (48.2)	0.693

All data are number (%).

*Family economic status: poor < 170 Afghanis (< 1.90 USD) per person per day; not poor ≥170 Afghanis (≥1.90 USD) per person per day.

^†^based on 25(OH)D level in the serum.

Table 3 shows the status of vitamin D in school children during the three seasons of the year. Data for summer were not available due to the school holidays. A statistically significant difference was not observed during the three seasons with sufficient (normal), insufficient, mild/moderate, and severe deficiency of vitamin D.

**Table 3:** Vitamin D status of the school children in different seasons of the year.

Vitamin D status	Total N = 510	Season of the year			*P*-value
		Autumn N = 163	Winter N = 177	Spring N = 170	
Sufficient (normal)	27 (5.3)	9 (33.3)	8 (29.6)	10 (37.1)	0.082
Insufficient	47 (9.2)	14 (29.8)	18 (38.3)	15 (31.9)	0.179
Mild/moderate deficiency	169 (33.1)	59 (34.9)	58 (34.3)	52 (30.8)	0.472
Severe deficiency	267 (52.4)	81 (30.4)	93 (34.8)	93 (34.8)	0.847

Data are number (%).

Univariate analysis (Table 4) using Chi-square test showed that the statistically significant risk factors associated with vitamin D deficiency in school children were: (i) age group 11–15 years (COR 2.9, 95% CI 1.3–6.6, *p* = 0.007), (ii) poverty (COR 2.3, 95% CI 1.1–4.8, *p* = 0.025), (iii) not doing outdoor physical activity (COR 4.6, 95% CI 2.8–7.8, and *p* = < 0.001), (iv) daily sunlight exposure < 60 min (COR 2.0, 95% CI 1.2–3.3, *p* = 0.006), and (v) not eating fish at least once every two weeks (COR 1.8, 95% CI 1.0–3.2, *p* = 0.049).

By logistic regression analysis, the statistically significant independent risk factors associated were: (i) age group 11–15 years (AOR 2.8, 95% CI 1.2–6.2, and *p* = 0.014), (ii) poverty (AOR 2.0, 95% CI 1.0–4.3, and p-value 0.049), (iii) not doing outdoor physical activity (AOR 4.8, 95% CI 2.8–8.1, and p-value < 0.001), and (iv) daily sunlight exposure < 60 min (AOR 2.2, 95% CI 1.3–3.7, and p-value 0.005).

**Table 4:** Univariate analyses and logistic regression examining factors associated with vitamin D deficiency among school children in Kandahar, Afghanistan.

Variable	Total N = 510	Vitamin D deficiency		COR (95% CI)	*P*-value	AOR (95% CI)*	*P*-value
		Present N = 436	Absent N = 74				
Age							
6–10 years 11–15 years	401 (78.6) 109 (21.4)	334 (83.3) 102 (93.6)	67 (16.7) 7 (6.4)	1 2.9 (1.3–6.6)	0.007	1 2.8 (1.2–6.2)	0.014
Gender							
Male Female	277 (54.3) 233 (45.7)	238 (85.9) 198 (85.0)	39 (14.1) 35 (15.0)	1 0.9 (0.6–1.5)	0.763	–	**–**
Mother literacy level							
Literate Illiterate	72 (14.1) 438 (85.9)	62 (86.1) 374 (85.4)	10 (13.9) 64 (14.6)	0.9 (0.5–1.9) 1	0.872	–	**–**
Father literacy							
Literate Illiterate	60 (11.8) 450 (88.2)	56 (93.3) 380 (84.4)	4 (6.7) 70 (15.6)	2.6 (0.9–7.3) 1	0.066	–	**–**
Ethnicity							
Pashtun Non-Pashtun	393 (77.1) 117 (22.9)	337 (85.8) 99 (84.6)	56 (14.2) 18 (15.4)	1.1 (0.6–1.9) 1	0.760	–	**–**
Family economic status^†^							
Poor Not poor	468 (91.8) 42 (8.2)	405 (86.5) 31 (73.8)	63 (13.5) 11 (26.2)	2.3 (1.1–4.8) 1	0.025	2.0 (1.0–4.3) 1	0.049
Obesity present							
Yes No	18 (3.5) 492 (96.5)	14 (77.8) 422 (85.8)	4 (22.2) 70 (14.2)	1 1.7 (0.6–5.4)	0.344	–	**–**
Doing outdoor physical activity							
Acceptable Unacceptable	166 (32.5) 344 (67.5)	119 (71.7) 317 (92.2)	47 (28.3) 27 (7.8)	1 4.6 (2.8–7.8)	< 0.001	1 4.8 (2.8–8.1)	< 0.001
Daily sunlight exposure							
≥60 min < 60 min	146 (28.6) 364 (71.4)	115 (78.8) 321 (88.2)	31 (21.2) 43 (11.8)	1 2.0 (1.2–3.3)	0.006	1 2.2 (1.3–3.7)	0.005
Topical use of sunscreen							
Yes No	32 (6.3) 478 (93.7)	14 (43.8) 211 (44.1)	18 (56.2) 267 (55.9)	1.1 (0.5–1.7) 1	0.714		
Eating fish (at least once in two weeks)							
Yes No	90 (17.6) 420 (82.4)	71 (78.9) 365 (86.9)	19 (21.1) 55 (13.1)	1 1.8 (1.0–3.2)	0.049	–	–

CI, confidence interval; COR, crude odds ratio; AOR, adjusted odds ratio.

*Variables not significant on bivariate analysis were not included in the logistic regression model.

^†^Family economic status: poor < 170 Afghanis (< 1.90 USD) per person per day; not poor ≥170 Afghanis (≥1.90 USD) per person per day.

## Discussion

In this school-based cross-sectional analytical study, we studied 510 primary school children for a period of seven months in six schools in Kandahar City, Afghanistan. We found a high prevalence of vitamin D deficiency in these school children and the associated risk factors were older age (11–15 years), decreased sunlight exposure, not doing outdoor physical activity, and poverty.

The prevalence of vitamin D deficiency in this study was a staggering 85%. Similar vitamin D deficiency prevalence rates have been reported from Tunisia, ~ 85% of 225 healthy children aged 7–16 years^26^, and Iran, ~ 80% in 477 healthy school children aged 9–18 years^27^. However, even higher rates have been observed in Saudi Arabia, 100% in 331 healthy urban children aged 6–17 years^28^, Malaysia, ~ 93% in 1361 healthy school students with a mean age of almost 13 years^29^, and India, 92% in 713 healthy school children aged 10–14 years^30^.

By contrast, lower but highly variable prevalence rates of vitamin D deficiency were been reported: no vitamin D deficiency in 103 healthy children (mean age 6.7 years)^31^, 3.6% in 225 healthy Tunisian children (mean age 9.5 years)^32^, 11.5% of 200 healthy primary school children, aged 9–11 years, from Egypt)^33^, 18% among 4,558 healthy American (USA) children aged 1–11 years^34^, almost 27% in 470 healthy Italian children aged 5–10 years^35^, ~ 30% in 435 healthy Algerian children aged 5–15 years^36^, ~ 35% in 1,102 healthy British children, aged 4–18 years^37^, ~ 40% in 99 healthy South Africans, aged 11–20 years^38^, 44% in 174 healthy Ethiopian students, aged 11–18 years^39^, and in Finland, 71% of 195 healthy school children and adolescents age 7–19 years^5^.

In all the above-mentioned studies, vitamin D deficiency was defined as a 25(OH)D concentration of < 50 nmol/L or < 20 ng/mL. The marked variation in vitamin D deficiency prevalence rates could be due to differences in various factors such as testing methods, latitudes, season of testing, age range tested, and nutritional habits. In North America and some European countries, vitamin D supplementation and fortification of foods with vitamin D is the main source of vitamin D^40,41^. However, this is not the case in Afghanistan where the dietary intake of vitamin D is poor, as fish and liver are scarce and relatively expensive^42^. Although better-off families can afford to eat a range of food, often they do not with one study reporting low food diversity scores throughout Afghanistan with an average of 15 (range 7–20)^42^. Furthermore, vitamin D-rich foods such as fish, liver, and eggs are believed to be “hot” or “hard” and are not given to children^14^.

In Kandahar, the cheapest way to obtain vitamin D is exposure to the sun. There is plenty of sunshine in Kandahar throughout the year and mean sun hours reported throughout the year range from 248 h in February to a high of 365 h in July for an annual mean of 329 h, which is higher than the mean sun hours for other large cities in Afghanistan^43^. Despite the long sun hours, the high prevalence of vitamin D deficiency in this study could be due to skin color (mostly brown), which negatively affects the synthesis of vitamin D by the skin, and traditional dress that covers the arms and legs. Therefore, the best options for the prevention of vitamin D deficiency in Afghan children could be health education regarding exposure to sunlight, as well as the provision of vitamin D supplements and vitamin D-fortified food.

In this study, being an older child (aged 11–15 years) was a risk factor for vitamin D deficiency, consistent with results from Iran (477 children aged 9–18 years)^27^, India (404 girls aged 6–18 years)^44^, Lebanon (179, aged 10–16 years)^45^, Turkey (51560, aged 0–18 years)^46^, Algeria (435, aged 5–15 years)^36^, and Great Britain (1102, aged 4–18 years)^37^. Compared to older children, younger children tend to be more physically active, spend more time outdoors (school and playground), and have a healthier diet enriched by dairy foods given to them by parents^47,48^. Puberty was identified as an independent factor in Iran due to the increased demand for vitamin D at this stage of development^27^. However, the Iranian study suggested more research was needed because further work is needed to explain the reasons for the decrease in serum 25(OH)D among older children since this appears to be independent of the effects of physical activity, pubertal status, sun exposure, or obesity^27^.

Two other key risk factors we identified that have also been reported widely across a range of studies from low to high-income countries were low outdoor physical activity and poverty^27,36,37,44,45,49^. Encouraging greater physical activity in prepubertal Caucasian children in Australia revealed that physical activity and sunlight exposure had a positive association with bone mass measurements and mineralization^50^ and should be included in health promotion activities. Tackling poverty is a greater challenge; foods that are rich in vitamin D, such as fish and liver, are expensive in Afghanistan^42^.

Being a girl was not a risk factor for vitamin D deficiency in this study and this accords with several other studies^27,37^ but thus has been reported as a factor in children from Malaysia (mean age ~ 13 years)^29^, Lebanon (10–16 years)^45^, Italy (5–10 years)^35^, and Finland (7–19 years)^5^. One factor in some societies may be the dress code for girls and women who are obliged to cover themselves; moreover, girls are more likely than boys to avoid the sun for cosmetic reasons.

### Strengths and limitations

This is the first study to examine vitamin D status among school children from Afghanistan. The sample size was large and covered six schools that were randomly selected throughout Kandahar City, allowing a degree of confidence in generalizing our results to Kandahar. The study had several limitations. First, it was only conducted in school-age children in Kandahar City; therefore, our results cannot be generalized to the whole country, different age groups, rural populations, and urban populations with different climates. Although we were not able to collect data during the summer, this is probably a minor limitation, given the high sunlight hours in Kandahar. The information for certain relied on memory; so, recall bias cannot be ruled out. Finally, this was a cross-sectional study, and the 25(OH)D concentration was only measured once; nevertheless, given the high rate of vitamin deficiency in these children, it is unlikely this had an impact on our results.

## Conclusion

We found a very high rate of vitamin D deficiency (> 85%) and more than half had severe deficiency even though Kandahar has abundant sunshine throughout the year. The main risk factor was poor physical activity followed by older age with similar risks for poverty and reduced sunshine exposure. Addressing vitamin D deficiency in Kandahar is not just about immediate health outcomes but ensuring a healthier future for the next generation of Afghanistan and avoiding the damaging effects of rickets.

Our results should be important and helpful to clinicians, nutritionists, public health officials, policymakers, parents, and government and non-government organizations who are engaged in the health and well-being of children in Afghanistan. We recommend that the Afghanistan Ministry of Public Health, with the help of international organizations (such as WFP, WHO, and UNICEF), develop strategies for enhancing nutritional education in schools, conducting periodic campaigns of vitamin D supplementation (aimed mostly at school children), encouraging and providing vitamin D-rich foods (e.g., oil, milk, and wheat flour), and promoting outdoor physical activities. Additional studies should be conducted on school children of rural areas in Kandahar province. Large-scale nationwide multicenter studies should be conducted across the age spectrum and including urban and rural populations to gain a better understanding of the challenge posed by vitamin D deficiency.

## Data Availability

All the data and materials related to this study are available on request from the corresponding author.

## Abbreviations

25: Hydroxyvitamin D25(OH)D
AOR: Adjusted odds ratio
BMI: Basal mass index
CI: Confidence interval
COR: Crude odds ratio
ng: Nanogram
mL: Milliliter
SD: Standard deviation
SPSS: Statistical Package for the Social Sciences
UNICEF: United Nations Children’s Fund
USD: United States Dollar
WFP: World Food Program
WHO: World Health Organization
